# Artificial genetic augmentation of T6SS activity affects effector repertoire and host toxicity

**DOI:** 10.1128/spectrum.01278-26

**Published:** 2026-10-15

**Authors:** Hadar Cohen, Eyal Elias, Dan Bar Yaacov, Dor Salomon, Motti Gerlic

**Affiliations:** 1 Gray School of Medical Sciences, Gray Faculty of Medical and Health Sciences, Tel Aviv University 26745, Tel Aviv, Israel; 2 The Shraga Segal Department of Microbiology, Immunology, and Genetics, Ben-Gurion University of the Negev 26732, Beer-Sheva, Israel; Universidad Andres Bello, Santiago, Chile

**Keywords:** T6SS, *Vibrio*, oyster, infection, toxicity, bacteria, effector, virulence

## Abstract

Type VI secretion system (T6SS), a toxic effector-delivery apparatus primarily studied for its antibacterial properties, has recently emerged as a widespread anti-eukaryotic determinant, particularly in members of the genus *Vibrio*. Although various anti-eukaryotic effectors have been described, it remains unknown whether effectors target distinct hosts in a similar manner. Moreover, it is unclear whether the entire effector arsenal encoded in a single genome is co-regulated across different conditions and by the same regulators. Here, we employ the anti-eukaryotic T6SS3 of *Vibrio proteolyticus* to address these knowledge gaps. By monitoring toxicity during infection of oyster hemocytes and comparing the results with previous infections of murine macrophages, we find that the effector Tie1 plays a role only in intoxicating murine cells, whereas Tie2 intoxicates both cell models. Furthermore, we show that artificial augmentation of T6SS3 activity by deleting the negative regulator *hns1* fails to induce the orphan effector Tie3, whereas overexpression of the T6SS3-specific activator, Ats3, induces all three known effectors. Using Ats3 overexpression to activate all three effectors, we show that both Tie2 and Tie3 participate in hemocyte intoxication. Collectively, these findings indicate that analyses of T6SS-mediated infections must factor in the possibility of partial activation of the effector repertoire even when the main gene cluster is fully induced, and that the contribution of anti-eukaryotic T6SS effectors to intoxication may be host-dependent.

**IMPORTANCE:**

The type VI secretion system (T6SS), a bacterial toxin-delivery apparatus, has recently been shown to mediate virulence in various marine *Vibrio* species. Each T6SS delivers a cocktail of toxins into a host cell, yet it is unknown whether toxins intoxicate different hosts in the same manner. Moreover, many investigations of T6SSs artificially activate the system by manipulating a transcription factor, yet it remains unknown whether different regulators induce the same toxin repertoire. Here, we show that a T6SS toxin that intoxicates one organism can be inert during the infection of another host. Furthermore, we demonstrate that T6SS-inducing regulators can activate distinct toxin repertoires. These findings suggest that conclusions about the role of T6SS toxins during infection should be drawn with caution, given the specific host model used, especially when the T6SS is artificially activated.

## OBSERVATION

Many Gram-negative bacteria deploy the type VI secretion system (T6SS), a toxic effector delivery apparatus, as an antibacterial tool to mediate interbacterial competition (1, 2). However, T6SSs are also known to target eukaryotes (3), and recent work has revealed several T6SS-mediated interactions with eukaryotes in marine *Vibrio* bacteria (4–10). One such example is T6SS3 in *Vibrio proteolyticus* (*Vpr*), which, as we have shown, is used to intoxicate the aquatic model organism *Artemia salina* (11). The activity of this T6SS also elicits inflammasome-dependent cytotoxicity in a mammalian immune cell model, murine bone marrow-derived macrophages (BMDMs) (12). *Vpr* T6SS3 secretes at least three anti-eukaryotic effectors: Tie1, Tie2, and Tie3 (loci ACWZF4_RS09470, ACWZF4_RS09465, and ACWZF4_RS22085, respectively). We previously established that Tie1 and Tie2, both encoded within the T6SS3 gene cluster, drive pyroptotic cell death in BMDMs (12), while the orphan effector Tie3, encoded outside the main T6SS cluster, works in concert with Tie1 and Tie2 to induce *Artemia* nauplii lethality (11).

In previous studies, we followed the methodology used in prior T6SS work (13–16) and manipulated positive (i.e., the T6SS3 regulator Ats3, a GlxA-family member encoded adjacent to the T6SS3 gene cluster [12]) or negative (i.e., the histone-like nucleoid-structuring repressor H-NS1, predicted to affect DNA condensation and block expression of horizontally acquired loci [17]) transcription regulators to augment T6SS3 activity and enhance T6SS-mediated toxic phenotypes (12). However, it remains unknown whether augmenting T6SS activity by manipulating different regulators differentially affects toxicity, and whether T6SS effectors have similar effects across distinct host models. To address these knowledge gaps, we set out to compare the effect of *Vpr* T6SS3 augmentation by genetically manipulating two distinct regulators during infection of immune cells (i.e., hemocytes) from a habitat-relevant marine organism, the oyster *Magallana gigas* (12). We used a strain with a deletion in the previously reported toxic hemolysin *vprh* (18) as the parental strain in this work, aiming to employ a reductionist approach and focus on T6SS3-mediated toxicity.

**Fig 1:**
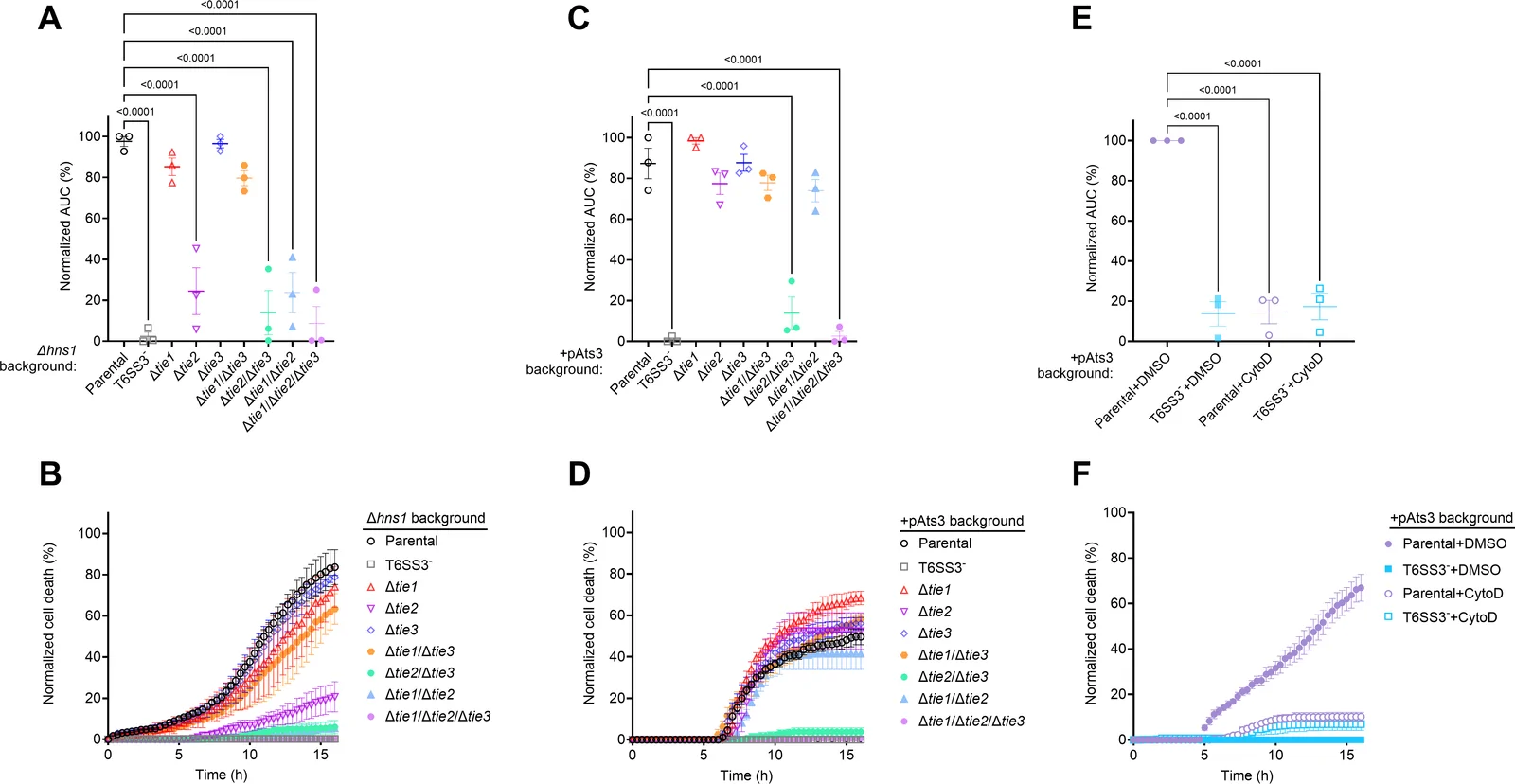
T6SS3 effector contribution to toxicity during oyster hemocyte infection depends on the inducing regulatory pathway. (**A–F**) Approximately 2 × 10^5^ hemocytes extracted from *M. gigas* hemolymph were seeded into 96-well plates coated with poly-D-lysine in Instant Ocean media in triplicate. Cells were infected with the indicated *Vpr* ∆*vprh* strains at a multiplicity of infection (MOI) = 5. Kanamycin was added to the media to maintain an arabinose-inducible plasmid for the expression of Ats3 (pAts3), and arabinose (0.1% [w/v]) was added to induce expression from the P*bad* promoter (**C–F**). When indicated (**E and F**), cytochalasin D (Cyto D; 5 µM) was added to the cells 30 min prior to infection; an equivalent volume of DMSO was added as a control. Propidium iodide (PI) uptake over 16 h was assessed by real-time microscopy (Incucyte SX5) as an indicator of cell death, and the data were recorded as PI-positive cells normalized to cell confluency at *t* = 0 h. The data (mean ± SD) of three independent experiments were then grouped as the area under the curve (AUC) (**A, C, and E**). The cell death kinetics of representative experiments are shown in panels B, D, and F (mean ± SE; *n* = 3 infection wells). Statistical comparisons in panels A, C, and E between samples at the 16-h timepoint were performed using a one-way ANOVA followed by Dunnett’s multiple comparison test; significant differences (*P* < 0.05) are denoted only for comparisons against the parental strain.

First, we used real-time microscopy (Incucyte) during hemocyte infection to monitor the toxicity of *Vpr* strains in which T6SS3 activity was augmented by deleting the negative regulator *hns1* (ACWZF4_RS09750). As we previously observed in BMDM infection (12), a Δ*hns1* strain induced dramatic hemocyte cell death that was completely dependent on T6SS3, as a T6SS3^−^ mutant strain (Δ*tssL3*) induced no cell death (Fig. 1A and B). Furthermore, the deletion of the effector *tie2*, alone or in combination with other effectors, was sufficient to abrogate toxicity in hemocytes. The complementation of H-NS1 from a plasmid restored T6SS3 toxicity levels to those of the parental strain (Fig. S1), supporting *hns1*’s role in regulating T6SS3 activity.

To further dissect the virulence activity of T6SS3 by engaging a different regulatory pathway, we infected oyster hemocytes with *Vpr* strains overexpressing Ats3. Real-time microscopy revealed that, as with *hns1* deletion, Ats3-overexpression induced a T6SS3-dependent cell death (Fig. 1C and D). This T6SS3-induced hemocyte cell death is dependent on bacterial internalization and was abolished in the presence of cytochalasin D, which inhibits phagocytosis by blocking actin polymerization (19, 20), as previously shown in BMDMs (12) (Fig. 1E and F). Surprisingly, in contrast to the reliance on Tie2 for hemocyte toxicity in strains in which T6SS3 is induced by *hns1* deletion, Ats3-induced T6SS3-dependent hemocyte cell death was not completely dependent on Tie2. Instead, the orphan effector Tie3 was sufficient to induce hemocyte cell death in the absence of Tie2, and only the deletion of both *tie2* and *tie3* abolished the T6SS3-dependent cell death (Fig. 1C and D). Together, these results imply that *hns1* deletion and Ats3 overexpression elicit hemocyte cell death that depends on distinct effector subsets. This observation prompted the hypothesis that the augmentation of T6SS3 by distinct transcriptional regulators includes distinct effector repertoires.

**Fig 2:**
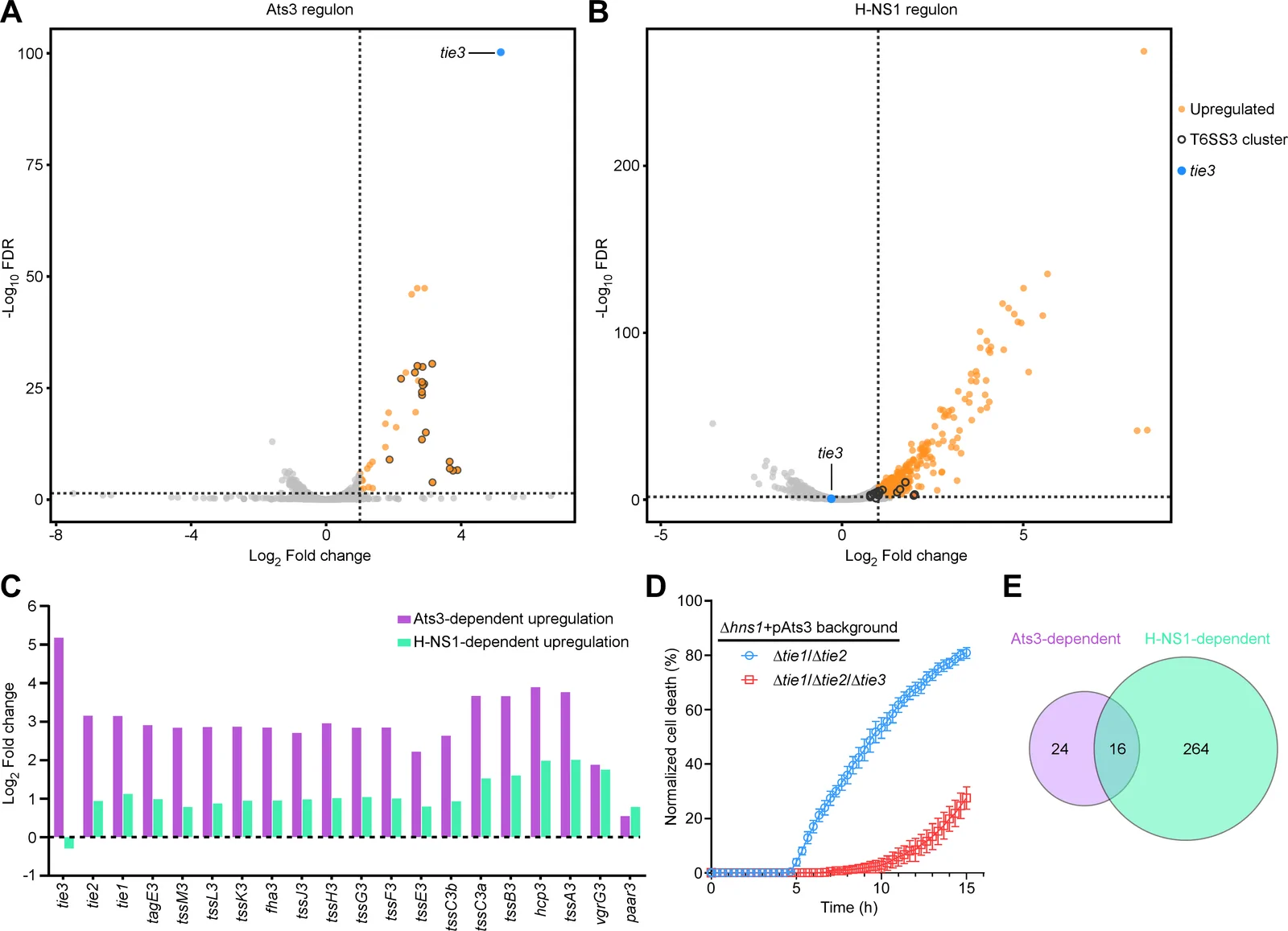
Ats3 overexpression, but not *hns1* deletion, upregulates *tie3* transcripts. (**A and B**) Volcano plots of the RNA-seq data are shown for wild-type *Vpr* overexpressing Ats3 from a plasmid (pAts3; **A**) or the Δ*hns1* derivative carrying an empty plasmid (pEmpty; **B**), each compared with wild-type *Vpr* carrying pEmpty. The artificially manipulated genes (*ats3* in A and *hns1* in B) were excluded from the plots for clarity. Significantly upregulated genes (orange) were considered as having a fold change > 2 and a false discovery rate (FDR) < 1%. (**C**) Comparison of T6SS3 gene expression changes in *Vpr* containing pAts3 or in a Δ*hns1* derivative containing pEmpty; fold change was calculated relative to wild-type *Vpr* containing pEmpty. (**D**) Approximately 2 × 10^5^ hemocytes extracted from *M. gigas* hemolymph were seeded into 96-well plates coated with poly-D-lysine in Instant Ocean media in triplicate. Cells were infected with the indicated *Vpr* ∆*vprh* strains at a multiplicity of infection (MOI) = 5. Kanamycin was added to the media to maintain an arabinose-inducible plasmid for the expression of Ats3 (pAts3), and arabinose (0.1% [w/v]) was added to induce expression from the P*bad* promoter. Propidium iodide (PI) uptake over 16 h was assessed by real-time microscopy (Incucyte SX5) as an indicator of cell death, and the data were recorded as PI-positive cells normalized to cell confluency at *t* = 0 h. Representative cell death kinetics from three independent experiments are shown (mean ± SE; *n* = 3 infection wells). (**E**) Venn diagram showing the number of genes significantly upregulated (fold change ≥ 2; FDR ≤ 0.01) by Ats3 overexpression and *hns1* deletion.

To test our hypothesis, we performed RNA-seq analyses and compared the effect of *hns1* deletion and Ats3 overexpression on the *Vpr* transcriptional landscape (Data set S1). Consistent with our hypothesis, the overexpression of Ats3 induced the expression of all known T6SS3 effectors, including the orphan effector *tie3*, whereas the deletion of *hns1* failed to induce *tie3* expression (Fig. 2A through C). Furthermore, we found that the overexpression of Ats3 in a ∆*hns1* background elicited *tie3*-dependent cell death in oyster hemocytes (Fig. 2D), indicating that the lack of *tie3* expression in the absence of *hns1* was not because of a mutation that impaired its expression potential. Thus, this difference in *tie3* expression accounts for the observed difference in its contribution to host toxicity between the *hns1* deletion and Ats3 overexpression strains. Although manipulation of both regulators upregulated the genes comprising the T6SS3 main cluster, Ats3 overexpression yielded a greater fold change than *hns1* deletion (Fig. 2C). Furthermore, *hns1* deletion resulted in a broad transcriptional reprogramming, affecting multiple pathways, including the upregulation of the antibacterial T6SS1 (Fig. 2B and E and Data set S2); in contrast, the Ats3 regulon was more specific to T6SS3 upregulation (Fig. 2A and E and Data set S3). Collectively, these results reveal upregulation of distinct T6SS3 effectors by distinct transcriptional cascades, thereby explaining the apparent differential reliance on specific effectors during host cell infection.

## CONCLUSIONS

In this work, we elucidate the role of *Vpr* T6SS3 during infection of immune cells from a marine animal model. Collectively, our past report on the toxic effects of this T6SS in murine macrophages (12) and our current results demonstrate that this T6SS can intoxicate immune cells from different host classes (i.e., Mammalia and Bivalvia). Although we previously showed that the effectors Tie1 and Tie2 promote BMDM cell death, here we demonstrate that the effectors Tie2 and Tie3, but not Tie1, are key mediators of hemocyte cell death. These differences imply that one’s conclusions about the contribution of specific effectors to virulence may depend on the host model selected for the assay. The distinct contribution of effectors to toxicity may stem from differences in the presence or expression levels of host targets between the host models, or from differences in the cellular immune response. Nevertheless, we cannot rule out the possibility that different media and temperature conditions, rather than different host models, contributed to the distinct effect of Tie1 during infection of BMDMs and oyster hemocytes.

In previous work, we demonstrated that the deletion of *hns* in *V. alginolyticus* activates all known T6SS1 effectors, including orphan effectors encoded outside the main T6SS cluster (13). In contrast, in *Vpr*, although *hns1* deletion upregulated T6SS3 activity and expression of effectors encoded within the main cluster, it did not induce the expression of the orphan effector *tie3 in vitro*. Notably, Tie3’s contribution to toxicity during the infection of *Artemia* nauplii with the ∆*hns1* strain (11), together with our findings that Ats3 overexpression elicits Tie3-dependent hemocyte cell death in a ∆*hns1* background, indicate that *tie3* induction is possible in this strain background, given Ats3-activating conditions found *in vivo*. These findings emphasize the critical role of activation mode in shaping the functional T6SS effector repertoire, particularly in systems harboring orphan effectors and in systems augmented by artificial regulator manipulations.

## MATERIALS AND METHODS

Detailed Materials and Methods are provided within the supplemental materials.

## Supplementary Material

Reviewer comments

## Data Availability

The *Vpr* genome assembly was deposited to NCBI under BioProject PRJNA1373524 (assembly GCA_053890595.1). RNA-seq data generated in this study were deposited in the NCBI SRA under accession PRJNA1422434.

## SUPPLEMENTAL MATERIAL

Data set S1 spectrum.01278-26-s0001.xlsx RNA-seq results.

Data set S2 spectrum.01278-26-s0002.xlsx Transcripts significantly upregulated upon hns1 deletion.

Data set S3 spectrum.01278-26-s0003.xlsx Transcripts significantly upregulated upon ats3 overexpression.

Supplemental materials spectrum.01278-26-s0004.pdf Supplemental methods, Fig. S1, Tables S1 and S2, Legends for Data sets S1 to S3, and supplemental references.

OPEN PEER REVIEW reviewer-comments.pdf An accounting of the reviewer comments and feedback.

ASM does not own the copyrights to Supplemental Material that may be linked to, or accessed through, an article. The authors have granted ASM a non-exclusive, world-wide license to publish the Supplemental Material files. Please contact the corresponding author directly for reuse.
