# Supplementary material for: Artificial genetic augmentation of T6SS activity affects effector repertoire and host toxicity
Source: Microbiol Spectr. 2026 Oct 15;14(11):e01278-26. doi: 10.1128/spectrum.01278-26 (PMC13729103; doi:10.1128/spectrum.01278-26)
Supplement: Supplementary file 1 [file reviewer-comments.pdf]

ASM | Microbiology Spectrum

Artificial genetic augmentation of T6SS activity affects effector repertoire and host toxicity

Hadar Cohen, Eyal Elias, Dan Bar Yaacov, Dor Salomon, and Mordechai Gerlic

Corresponding Author(s): Dor Salomon, Tel Aviv University

**Review Timeline:**		
	Submission Date:	April 14, 2026
	Editorial Decision:	June 23, 2026
	Revision Received:	September 22, 2026
	Accepted:	September 23, 2026

Editor: Carlos Blondel

Reviewer(s): The reviewers have opted to remain anonymous.

Transaction Report:

(Note: With the exception of the correction of typographical or spelling errors that could be a source of ambiguity, letters and reports are not edited. The original formatting of letters and referee reports may not be reflected in this compilation.)

DOI: https://doi.org/10.1128/spectrum.01278-26

Re: Spectrum01278-26 (**Choice of host model and manipulated transcription regulator dictates T6SS effector-mediated toxicity**)

Dear Prof. Dor Salomon:

Thank you for the privilege of reviewing your work. Below you will find my comments, instructions from the Spectrum editorial office, and the reviewer comments.

Of note, both reviewers have highlighted the importance of performing complementation assays to further support the work's conclusions. In addition, I believe most of the other comments raised by the reviewers can be addressed by rephrasing or softening certain claims and statements.

Please return the manuscript within 60 days; if you cannot complete the modification within this time period, please contact me. If you do not wish to modify the manuscript and prefer to submit it to another journal, notify me immediately so that the manuscript may be formally withdrawn from consideration by Spectrum.

Revision Guidelines
To submit your modified manuscript, log into the submission site at https://spectrum.msubmit.net/cgi-bin/main.plex. Go to Author Tasks and click the appropriate manuscript title to begin. The information you entered when you first submitted the paper will be displayed; update this as necessary. Note the following requirements:

• Upload point-by-point responses to the issues raised by the reviewers in a file named "Response to Reviewers," NOT in your cover letter.
• Upload a compare copy of the manuscript (without figures) as a "Marked-Up Manuscript" file.
• Upload a clean .DOC/.DOCX version of the revised manuscript and remove the previous version.
• Each figure must be uploaded as a separate, editable, high-resolution file (TIFF or EPS preferred), and any multipanel figures must be assembled into one file.
• Any supplemental material intended for posting by ASM should be uploaded with their legends separate from the main manuscript. You can combine all supplemental material into one file (preferred) or split it into a maximum of 10 files with all associated legends included.

For complete guidelines on revision requirements, see our Submission and Review Process webpage. Submission of a paper that does not conform to guidelines may delay acceptance of your manuscript.

Data availability: ASM policy requires that data be available to the public upon online posting of the article, so please verify all links to sequence records, if present, and make sure that each number retrieves the full record of the data. If a new accession number is not linked or a link is broken, provide Spectrum production staff with the correct URL for the record. If the accession numbers for new data are not publicly accessible before the expected online posting of the article, publication may be delayed; please contact production staff (Spectrum@asmusa.org) immediately with the expected release date.

Publication Fees: For information on publication fees and which article types are subject to charges, visit our website. If your manuscript is accepted for publication and any fees apply, you will be contacted separately about payment during the production process; please follow the instructions in that e-mail. Arrangements for payment must be made before your article is published.

ASM Membership: Corresponding authors may join or renew ASM membership to obtain discounts on publication fees. Need to upgrade your membership level? Please contact Customer Service at Service@asmusa.org.

The ASM Journals program strives for constant improvement in our submission and publication process. Please tell us how we can improve your experience by taking this quick Author Survey.

Thank you for submitting your paper to Spectrum.

Sincerely,
Carlos Blondel
Editor
Microbiology Spectrum

Reviewer #1 (Comments for the Author):

This manuscript presents experiments to address specific questions relating to effector repertoires in a pathogenic Vibrio strain.

The authors take advantage of a mutant background (hns deletion) along with a overexpression system to augment the cytotoxicity phenotypes observed during infection. Based on the bacterial strain systems and contexts, the conclusions are clear and readily observable. The main issues are that the experimental systems, specifically the strain backgrounds, are very different and likely have very different physiological outcomes. There is a concern that hns mutants are highly deregulated across the entire genome. Moreover, such strains are somewhat genetically unstable and usually develop secondary mutations that are not easily identified without sequencing. While the use of single, double, and triple mutants is necessary, the approach should be taken with caution. Without data for genetic complementation, the experiments should be interpreted with caution.

Comments for consideration:
1. It would be worthwhile to genetically complement the hns mutant to show it can be returned to the parental (or wild type) strain phenotype. On this topic, are all strains VprH deficient (including the parental strain)?
2. What happens if the hns mutant were to overexpress Ats3? This is important because hns mutants are typically de-repressed (not activated). In contrast, Ats3 likely and specifically activates genes in a transcription favoring manner. The issue here is that not all genomic loci are de-repressed in a similar manner within hns mutants. H-NS is involved in constraining negative supercoiling and works in context of other nucleoid proteins.
3. Line 104-106. I think this is testable!? Is it possible to generate a plasmid that expresses Tie3 (inducible or constitutive) and then measure host toxicity? I think this would be a great experiment to strengthen the manuscript.
Minor point
1. There should be some statement of caution in the text relating to the experimental system and strain backgrounds. As most of the data experiments do not have the wild type (no mutations) strain, the context does matter.
2. Another interpretation is that the host targets in each cell system (murine macrophage and hemocytes) are expressed at different levels. In such a scenario, the effect of specific T6SS effectors would be expected to be different.

Reviewer #2 (Comments for the Author):

In this work, Cohen et al describe experiments using hemocytes from an oyster model, which are treated with several presumably isogenic isolates of Vibrio parahaemolyticus carrying deletions is two regulatory genes and in three T6SS effectors. The authors observe cytotoxicity that varies according to the genetic modifications of the Vp isolates and conclude that distinct regulation and the choice of host model dictates effector-mediate cytotoxicity, as indicated in the title.

I have several major concerns with this work.

1. The title implies that it is the "choice of model" that determines effector-mediate cytotoxicity. I would think the T6SS has co-evolved with the microorganism in its natural habitat(s); hence, the differences in cytotoxicity will vary in relation to natural selection. The choice of host is artificial, and human based. The same consideration applies to "manipulation of transcription regulators. This is also artificial. I would argue that in vivo, several regulators will contribute to the outcome of infection by Vp. In my opinion, the title is misleading and needs to be revised.

2. The notion that the T6SS acting on different host cells (oyster's hemocytes and mouse BMDMs) relies on historical results. There are no data presented in which cells from both models were compared side by side with the mutants in this study. Making historical comparisons is dangerous and potentially flawed. At best, the authors can comment on the differences between models in the conclusions, based on existing literature, but not in the body of the results. This work only shows that two different regulators are associated to the expression of different T6SS effectors using the oyster model system!

3. The manuscript lacks explanations that are key to understand the experiments and the overall experimental strategy. It is unclear what type of regulator AtsA is. We know that the hns is a global regulator that results in pleiotropic phenotypes; its regulation mechanism, influencing DNA bending, makes it also dependent on DNA local conditions across the chromosome. Therefore, it is not surprising that will not regulate every possible gene related to T6SS, especially is Tai3 is encoded outside the main cluster. As for AtsA, its effect is manifested by overexpression; this is artificial. The authors have not shown any evidence that the effect of the regulators is direct on the concerned genes. Is it any evidence that AtsA regulates these genes when the regulator is expressed from the chromosome? Explaining what AtsA is and how does it work would help understand this better.

4. The authors claim the strains are isogenic, but they are dealing with deletion mutants. I do not know about astA, but hns can cause pleiotropism. Complementation of the phenotypes should deal with this. Also, it is unclear if the authors have used a WT Wv carrying the empty vector to rule out any effects from the plasmid vector and the antibiotic pressure on the experiments.

5. The body of the work falls mainly on Fig. 1. The results are presented as %AUC. Also, the results are expressed as SEM (from n=3) which reduces the variance. SD would be preferably to report the actual variance across experiments. Alternative, it would better to express the variance as % confidence intervals. This becomes relevant in the results where combination of mutants gives apparent non-significant differences but differ in more and 20%AUC. Are these curves the ones presented in panels B, D, F?

Moreover, one would assume that there will be growth differences of the mutants, especially in the regulator deletions. Has this been tested?
Finally, have the authors included a WT with the empty vector as a control (panels E-F)?

6. Fig 2. The title is misleading because the authors are comparing the overexpression of one regulator and the deletion of another. Also, can they comment on whether hns1 does regulate Ats3? What are the effects of the regulators, once manipulated, on bacterial growth/fitness in general?

7. There are several sentences that require attention by the authors since they are misleading and confusing. For example:

a. L50-52. Cryptic sentence; what is the underappreciated role of the T6SS in eukaryotes? Not only the sentence is incomprehensible, but also misleading. The effect of T5SS on eukaryotic has been shown for multiple cells including fungi. The authors, only refer mostly their work or work on Vp.

b. L52-55. Confusing. It reads as it the bacterium is used to intoxicate and induce inflammation. But is the other way around. The model is used to test if the bacterium can cause any harm

c. L62, delete "we drew...T6SS works"

d. L65-68. Misleading. T6SS in general are regulated in multiple ways involving both transcriptional and post-transcriptional factors. Also, T6SS clusters in many cases have a mosaic structure with several transcriptional units (mini operons). These notions may or may not apply to the Vp system but should be explained to place this research in context with the literature in the field.

e. L72. The results are presented as fluorescent units. There is no microscopy

f. L73-74. Presumably the regulator was not deleted during infection. The infection was investigated with a previously made deletion mutant; right?

g. L80-82. This sentence needs work too since it is unclear and again, misleading. This is rather a general conclusion that should be presented below. The experiments shown in this paper are about regulators and a set of T6SS effectors in one model system.

h. Please delete amplifiers like "surprisingly", "notably", "interestingly", "pivotal", "completely", etc. The sentences look better without them.

g. L112-114. This conclusion needs references. Do the authors really think that this conclusion is novel or unique?

i. L136-138. This needs further thought, as it would apply to many orphan effectors related to antibacterial competition, which are not expressed or utilized in the usual prey models that are used. In essence, we can only see what we can find, and what we can find depends on the model we use. This is, I think, the point the authors want to make. However, while a valuable addition to the field, this is not a new concept.

Reviewer #1 (Comments for the Author):

This manuscript presents experiments to address specific questions relating to effector repertoires in a pathogenic Vibrio strain. The authors take advantage of a mutant background (hns deletion) along with a overexpression system to augment the cytotoxicity phenotypes observed during infection. Based on the bacterial strain systems and contexts, the conclusions are clear and readily observable. The main issues are that the experimental systems, specifically the strain backgrounds, are very different and likely have very different physiological outcomes. There is a concern that hns mutants are highly deregulated across the entire genome. Moreover, such strains are somewhat genetically unstable and usually develop secondary mutations that are not easily identified without sequencing. While the use of single, double, and triple mutants is necessary, the approach should be taken with caution. Without data for genetic complementation, the experiments should be interpreted with caution.

We thank the reviewer for their input. We agree that caution is warranted when analyzing the results (this is a main conclusion of this work), and we have added control experiments and revised the text to support or soften our claims.

Comments for consideration:

1. It would be worthwhile to genetically complement the hns mutant to show it can be returned to the parental (or wild type) strain phenotype.

As suggested by the reviewer, we now show that the expression of H-NS1 from a plasmid in the ∆hns1 background strain suppresses T6SS3 activity, as evidenced by reduced toxicity during oyster hemocyte infection (Supplementary Figure S1 and lines 84-86).

On this topic, are all strains VprH deficient (including the parental strain)?

Yes, all the strains used in the infection assays contain a vprh deletion. The reasoning behind this is our aim to focus on T6SS-mediated toxicity while eliminating other known virulence factors. This is mentioned in the relevant Figure legends, and now also in the main text (lines 73-76): "We used a strain with a deletion in the previously reported toxic hemolysin vprh (18) as the parental strain in this work, aiming to employ a reductionist approach and focus on T6SS3-mediated toxicity".

2. What happens if the hns mutant were to overexpress Ats3? This is important because hns mutants are typically de-repressed (not activated). In contrast, Ats3 likely and specifically activates genes in a transcription favoring manner. The issue here is that not all genomic loci are de-repressed in a similar manner within hns mutants. H-NS is involved in constraining negative supercoiling and works in context of other nucleoid proteins.

We thank the reviewer for raising this point, because this is exactly what this work aims to convey. We are experimentally demonstrating that artificial augmentation of

T6SS activity by manipulating a transcription regulator has distinct effects on effector expression, thus affecting the toxic outcomes and the conclusions drawn from the experiments.
We now include results showing that overexpression of Ats3 in the hns1-deletion background elicits tie3-dependent hemocyte cell death, indicating that: (i) the reason for the lack of Tie3 contribution to cell death in the hns1 strain is not an inactivating mutation in tie3, and (ii) that Ats3 is required to induce tie3 expression (Figure 2D, lines 108-111).

3. Line 104-106. I think this is testable!? Is it possible to generate a plasmid that expresses Tie3 (inducible or constitutive) and then measure host toxicity? I think this would be a great experiment to strengthen the manuscript.

We thank the reviewer for this suggestion. As mentioned in the previous comment, we now show that Ats3 expression elicits a tie3-dependent cell death in the ∆hns1/∆tie1/∆tie2 background (Figure 2D), supporting the hypothesis that "the difference in tie3 expression accounts for the observed difference in its contribution to host toxicity between the hns1 deletion and Ats3 overexpression strains".

Minor point
1. There should be some statement of caution in the text relating to the experimental system and strain backgrounds. As most of the data experiments do not have the wild type (no mutations) strain, the context does matter.

We have added a note explaining the reasoning behind using the vprh mutant background (lines 73-76).

2. Another interpretation is that the host targets in each cell system (murine macrophage and hemocytes) are expressed at different levels. In such a scenario, the effect of specific T6SS effectors would be expected to be different.

We agree with the reviewer that this is a possible explanation of the observed phenotypes. To clarify this point, we now mention in the Conclusions section that (Lines 133-138): "The distinct contribution of effectors to toxicity may stem from differences in the presence or expression levels of host targets between the host models, or from differences in the cellular immune response. Nevertheless, we cannot rule out the possibility that different media and temperature conditions, rather than different host models, contributed to the distinct effect of Tie1 during infection of BMDMs and oyster hemocytes".

Reviewer #2 (Comments for the Author):

In this work, Cohen et al describe experiments using hemocytes from an oyster model, which are treated with several presumably isogenic isolates of Vibrio parahaemolyticus carrying deletions is two regulatory genes and in three T6SS effectors. The authors observe cytotoxicity that varies according to the genetic modifications of the Vp isolates and conclude that distinct regulation and the choice of host model dictates effector-mediate cytotoxicity, as indicated in the title.

I have several major concerns with this work.

1. The title implies that it is the "choice of model" that determines effector-mediate cytotoxicity. I would think the T6SS has co-evolved with the microorganism in its natural habitat(s); hence, the differences in cytotoxicity will vary in relation to natural selection. The choice of host is artificial, and human based. The same consideration applies to "manipulation of transcription regulators. This is also artificial. I would argue that in vivo, several regulators will contribute to the outcome of infection by Vp. In my opinion, the title is misleading and needs to be revised.

We agree with the reviewer, as this analysis precisely echoes our conclusions. The conclusion of this work concerns artificial experiments performed in the lab using genetically manipulated bacterial strains and a chosen host model, rather than a natural infection process. We demonstrate that when one selects a particular host model, and/or genetically manipulates the bacterial strain to augment T6SS activity (as previously done by various labs across different bacteria, including in our own work), these choices may affect conclusions regarding the contribution of specific effectors. To better convey this message, we revised the title to: "Artificial genetic augmentation of T6SS activity affects effector repertoire and host toxicity".

2. The notion that the T6SS acting on different host cells (oyster's hemocytes and mouse BMDMs) relies on historical results. There are no data presented in which cells from both models were compared side by side with the mutants in this study. Making historical comparisons is dangerous and potentially flawed. At best, the authors can comment on the differences between models in the conclusions, based on existing literature, but not in the body of the results. This work only shows that two different regulators are associated to the expression of different T6SS effectors using the oyster model system!

Following the reviewer's comment, we removed the sentence regarding Tie1's effects on different host cells from the main text. This is now only discussed in the Conclusions section. Nevertheless, we wish to emphasize that our qualitative comparisons are based on our own published work, conducted with the same strains and personnel as in the current report.

3. The manuscript lacks explanations that are key to understand the experiments and the overall experimental strategy. It is unclear what type of regulator AtsA is. We know that the hns is a global regulator that results in pleiotropic phenotypes; its

regulation mechanism, influencing DNA bending, makes it also dependent on DNA local conditions across the chromosome. Therefore, it is not surprising that will not regulate every possible gene related to T6SS, especially is Tai3 is encoded outside the main cluster. As for AtsA, its effect is manifested by overexpression; this is artificial. The authors have not shown any evidence that the effect of the regulators is direct on the concerned genes. Is it any evidence that AtsA regulates these genes when the regulator is expressed from the chromosome? Explaining what AtsA is and how does it work would help understand this better.

Regarding Ats3, no experimental data are available beyond our previous work showing that overexpression of this gene, which is naturally located adjacent to the T6SS3 cluster, upregulates T6SS3 but not T6SS1 (Cohen et al., eLife, 2022). Ats3 contains a GlxA domain (COG4977), predicted to contain an amidase domain and an AraC-type DNA-binding HTH domain. We have added this information, along with information on H-NS, to the text (lines 62-67): "In previous studies, we followed the methodology used in prior T6SS work (13–16) and manipulated positive (i.e., the T6SS3 regulator Ats3, a GlxA-family member encoded adjacent to the T6SS3 gene cluster (12)) or negative (i.e., the histone-like nucleoid-structuring repressor H-NS1, predicted to affect DNA condensation and block expression of horizontally acquired loci (17)) transcription regulators to augment T6SS3 activity and enhance T6SS-mediated toxic phenotypes (12)".

In the revised version of the manuscript, we further clarify the objective and approach used in this study (lines 70-73): "To address these knowledge gaps, we set out to compare the effect of Vpr T6SS3 augmentation by genetically manipulating two distinct regulators during infection of immune cells". As the reviewer correctly states, the effects observed by these genetic manipulations are artificial, as they are in other articles that employ a similar genetic approach to augment and investigate T6SSs. Indeed, this is the point that we wish to emphasize in this Observation – that the nature of a genetic manipulation employed to artificially augment T6SS activity matters.

4. The authors claim the strains are isogenic, but they are dealing with deletion mutants. I do not know about astA, but hns can cause pleiotropism. Complementation of the phenotypes should deal with this.

We have removed the word "isogenic" from the text.

In addition, we now show that the expression of H-NS1 from a plasmid in the ∆hns1 background strain suppresses T6SS3 activity, as evidenced by reduced toxicity during oyster hemocyte infection (Supplementary Figure S1 and lines 84-86).

Also, it is unclear if the authors have used a WT Wv carrying the empty vector to rule out any effects from the plasmid vector and the antibiotic pressure on the experiments.

In all experiments involving a plasmid, all tested strains contain one. In the infection assays, all strains carry the plasmid expressing Ats3 (pAts3), as detailed in the

figures. In the RNA-seq experiments, strains contain either pAts3 or an empty plasmid (pEmpty), as detailed in the Figure 2 caption.

5. The body of the work falls mainly on Fig. 1. The results are presented as %AUC. Also, the results are expressed as SEM (from n=3) which reduces the variance. SD would be preferably to report the actual variance across experiments. Alternative, it would better to express the variance as % confidence intervals. This becomes relevant in the results where combination of mutants gives apparent non-significant differences but differ in more and 20%AUC. Are these curves the ones presented in panels B, D, F?

The results in Figure 1 are presented both as %AUC from three independent biological experiments, each performed in triplicate (panels A, C, E), and as normalized % cell death from one representative experiment (panels B, D, F), which is included in the %AUC panels, as stated in the figure caption. By "reduces the variance", we assume the reviewer does not mean the actual variance of the data, which remains unaffected by the analysis, but rather the appearance of the error bars. Notably, the primary cells used in each experiment were pooled from 3 to 10 individual oysters (as clarified in the supplementary Methods section) to minimize noise and create a standardized, population-representing sample. Presenting these curves as mean ± SEM (from pseudo-biological triplicates) demonstrates the high technical precision and stability of this profile over time, and is a well-known practice in similar experimental settings. Furthermore, we provide the individual sample values for all the graphs in panels A, C, and E, allowing the reader to visualize the true variance between experiments. In the AUC panels, we present independent analyses of three distinct pools of oyster hemocytes (each representing 24 to 30 individual oysters). Because the starting material for each experiment is already a pooled cell sample, SEM is methodologically appropriate for capturing the precision of our final population mean estimate, rather than the raw variance of individual oysters.

Moreover, one would assume that there will be growth differences of the mutants, especially in the regulator deletions. Has this been tested?

An assay showing similar growth between the same hns1-deletion strains used here was performed and published in our previous paper (Cohen et al., Microbiology Spectrum, 2023; PMID: 37310285, Figure S4).

Finally, have the authors included a WT with the empty vector as a control (panels E-F)?

A WT strain was not used here; instead, a vprh-deleted strain was used as the parental strain throughout the work. The reasoning for that is now explained in the text (lines 73-76): "We used a strain with a deletion in the previously reported toxic hemolysin vprh (18) as the parental strain in this work, aiming to employ a reductionist approach and focus on T6SS3-mediated toxicity".

Furthermore, all the strains tested in this assay contain a plasmid, as detailed in the figure.

6. Fig 2. The title is misleading because the authors are comparing the overexpression of one regulator and the deletion of another.

Please note that the comparisons shown in Figure 2 are not directly between a strain over-expressing Ats3 and a strain containing an hns1 deletion. Rather, each strain is compared separately to the WT strain, and the conclusion of the tie3 induction is made on the basis of these separate comparisons.

Also, can they comment on whether hns1 does regulate Ats3?

Our RNA-seq results do not reveal a significant up- or down-regulation of ats3 (ACWZF4_RS09460) by H-NS1 (see Supplementary Dataset S2).

What are the effects of the regulators, once manipulated, on bacterial growth/fitness in general?

We previously reported that hns1 deletion has a minimal effect on growth in certain media and no significant effect on swimming, whereas Ats3 overexpression has no effect on either growth or swimming (Cohen et al., eLife, 2022). However, please note that we do not make a direct, quantitative comparison between the hns1-deleted strain and the strain overexpressing Ats3.

7. There are several sentences that require attention by the authors since they are misleading and confusing. For example:

a. L50-52. Cryptic sentence; what is the underappreciated role of the T6SS in eukaryotes? Not only the sentence is incomprehensible, but also misleading. The effect of T5SS on eukaryotic has been shown for multiple cells including fungi. The authors, only refer mostly their work or work on Vp.

Following the reviewer's comment, the sentence was revised to: "However, T6SSs are also known to target eukaryotes (3), and recent work has revealed several T6SS-mediated interactions with eukaryotes in marine Vibrio bacteria (4–10)". Additional references were added.

b. L52-55. Confusing. It reads as it the bacterium is used to intoxicate and induce inflammation. But is the other way around. The model is used to test if the bacterium can cause any harm

We clarified the sentence by splitting it into two. It now reads (lines 52-56): "One such example is T6SS3 in Vibrio proteolyticus (Vpr), which, as we have shown, is used to intoxicate the aquatic model organism Artemia salina (11). The activity of this T6SS also elicits inflammasome-dependent cytotoxicity in a mammalian immune cell model, murine bone marrow-derived macrophages (BMDMs) (12)".

c. L62, delete "we drew...T6SS works"

We revised the sentence (lines 62-67): "In previous studies, we followed the methodology used in prior T6SS work…".

d. L65-68. Misleading. T6SS in general are regulated in multiple ways involving both transcriptional and post-transcriptional factors. Also, T6SS clusters in many cases have a mosaic structure with several transcriptional units (mini operons). These notions may or may not apply to the Vp system but should be explained to place this research in context with the literature in the field.

We have revised the sentence for clarity. It now reads (lines 68-70): "However, it remains unknown whether augmenting T6SS activity by manipulating different regulators differentially affects toxicity, and whether T6SS effectors have similar effects across distinct host models".

We agree with the reviewer's comment that T6SSs have diverse layers and regulatory mechanisms across various bacteria. Nevertheless, this is not the focus of this work, which is that artificial regulator manipulation should be used with caution, as different regulatory pathways may induce distinct effector repertoires.

e. L72. The results are presented as fluorescent units. There is no microscopy

The data are presented as the percentage of cell death, derived from fluorescence measurements. The data were acquired using an Incucyte, which is a microscope housed in a temperature-controlled incubator. As described in the supplementary Methods section, cell death is quantified via propidium iodide fluorescent signal in each acquired microscope image.

f. L73-74. Presumably the regulator was not deleted during infection. The infection was investigated with a previously made deletion mutant; right?

We revised the sentence for clarity. It now reads (lines 77-79): "First, we used real-time microscopy (Incucyte) during hemocyte infection to monitor the toxicity of Vpr strains in which T6SS3 activity was augmented by deleting the negative regulator hns1 (ACWZF4_RS09750)".

g. L80-82. This sentence needs work too since it is unclear and again, misleading. This is rather a general conclusion that should be presented below. The experiments shown in this paper are about regulators and a set of T6SS effectors in one model system.

The sentence has been removed in the revised manuscript.

h. Please delete amplifiers like "surprisingly", "notably", "interestingly", "pivotal", "completely", etc. The sentences look better without them.

We removed most of these words from the text.

g. L112-114. This conclusion needs references. Do the authors really think that this conclusion is novel or unique?

Respectfully, we are puzzled by this comment. The conclusion specifically refers to T6SS3 in V. proteolyticus. It has not been previously published and is based on the data presented in this work. Whether the concept is unique or not is irrelevant.

i. L136-138. This needs further thought, as it would apply to many orphan effectors related to antibacterial competition, which are not expressed or utilized in the usual prey models that are used. In essence, we can only see what we can find, and what we can find depends on the model we use. This is, I think, the point the authors want to make. However, while a valuable addition to the field, this is not a new concept.

This is not exactly the point we are trying to make. We propose that the experimental setup, especially if it includes T6SS activity augmentation via genetic manipulations, can lead to the expression of different effector sets, thus requiring caution when analyzing results and assigning roles to specific effectors. We are unaware of works that have experimentally demonstrated this concept and would appreciate it if the reviewer could point us to such reports so we can discuss and cite them further.

Re: Spectrum01278-26R1 (**Artificial genetic augmentation of T6SS activity affects effector repertoire and host toxicity**)

Dear Prof. Dor Salomon:

Your manuscript has been accepted, and I am forwarding it to the ASM production staff for publication. Your paper will first be checked to make sure all elements meet the technical requirements. ASM staff will contact you if anything needs to be revised before copyediting and production can begin. Otherwise, you will be notified when your proofs are ready to be viewed.

Data Availability: ASM policy requires that data be available to the public upon online posting of the article, so please verify all links to sequence records, if present, and make sure that each number retrieves the full record of the data. If a new accession number is not linked or a link is broken, provide production staff with the correct URL for the record. If the accession numbers for new data are not publicly accessible before the expected online posting of the article, publication may be delayed; please contact ASM production staff immediately with the expected release date.

Publication Fees: For information on publication fees and which article types have charges, please visit our website. If your article is accepted and publication fees apply, you will receive an email from CCC/RightsLink, ASM's payment partner, with a link to their secure portal to complete your payment. ASM will never ask you to provide payment details, banking information, or credit card information by email. If you receive an email requesting payment information directly, do not respond. You may contact ASM at any time for verification of any payment-related messages you receive. Note that payment must be received before your article is published.

ASM Membership: Corresponding authors may join or renew ASM membership to obtain discounts on publication fees. Need to upgrade your membership level? Please contact Customer Service at Service@asmusa.org.

Embargo Policy: A press release may be issued as soon as the manuscript is posted on the Spectrum Latest Articles webpage. The corresponding author will receive an email with the subject line "ASM Journals Author Services Notification" when the article is available online.

Cover Image Submissions: If you would like to submit a potential Cover Image, please email a file and a short legend to spectrum@asmusa.org. Please note that we can only consider images that (i) the authors created or own and (ii) have not been previously published. By submitting, you agree that the image can be used under the same terms as the published article. Image File requirements: TIF/EPS, 7.5 inches wide by 8.25 inches tall (at least 2,250 pixels wide by 2,475 pixels tall), minimum 300 dpi resolution (600 dpi preferred), RGB, and no figure elements, e.g., arrows or panel labels. The legend should be a short description of the image, 1-2 sentences recommended. Please use the ASM Journals Cover Image Sizing Tool (https://journals.asm.org/pb-assets/html/asm-cover-sizing-tool.html) to ensure that your proposed cover image meets our size requirements.

The ASM Journals program strives for constant improvement in our submission and publication process. Please tell us how we can improve your experience by taking this quick Author Survey.

Thank you for submitting your paper to Spectrum.

Sincerely,
Carlos Blondel
Editor
Microbiology Spectrum
